# Seafloor heterogeneity influences the biodiversity–ecosystem functioning relationships in the deep sea

**DOI:** 10.1038/srep26352

**Published:** 2016-05-23

**Authors:** Daniela Zeppilli, Antonio Pusceddu, Fabio Trincardi, Roberto Danovaro

**Affiliations:** 1 Department of Life and Environmental Sciences, Università Politecnica delle Marche, Via Brecce Bianche, 60131 Ancona, Italy; 2 IFREMER, Centre Brest, REM/EEP/LEP, Institut Carnot Ifremer-EDROME, ZI de la pointe du diable, CS10070, F-29280 Plouzané, France; 3 Department of Life and Environmental Sciences, University of Cagliari, Via Fiorelli 1, 09126 Cagliari, Italy; 4 ISMAR (CNR), via Gobetti 101, 40129 Bologna, Italy; 5 Stazione Zoologica Anton Dohrn, Villa Comunale, 80121, Naples, Italy

## Abstract

Theoretical ecology predicts that heterogeneous habitats allow more species to co-exist in a given area. In the deep sea, biodiversity is positively linked with ecosystem functioning, suggesting that deep-seabed heterogeneity could influence ecosystem functions and the relationships between biodiversity and ecosystem functioning (BEF). To shed light on the BEF relationships in a heterogeneous deep seabed, we investigated variations in meiofaunal biodiversity, biomass and ecosystem efficiency within and among different seabed morphologies (e.g., furrows, erosional troughs, sediment waves and other depositional structures, landslide scars and deposits) in a narrow geo-morphologically articulated sector of the Adriatic Sea. We show that distinct seafloor morphologies are characterized by highly diverse nematode assemblages, whereas areas sharing similar seabed morphologies host similar nematode assemblages. BEF relationships are consistently positive across the entire region, but different seabed morphologies are characterised by different slope coefficients of the relationship. Our results suggest that seafloor heterogeneity, allowing diversified assemblages across different habitats, increases diversity and influence ecosystem processes at the regional scale, and BEF relationships at smaller spatial scales. We conclude that high-resolution seabed mapping and a detailed analysis of the species distribution at the habitat scale are crucial for improving management of goods and services delivered by deep-sea ecosystems.

One of the emerging aims of marine ecology is to better understand which processes produce and maintain biodiversity in the deep sea and at which spatial scales these processes operate^1^,^2^,^3^,^4^,^5^,^6^,^7^. Spatial heterogeneity influences at various scales important ecosystem features including populations’ structure, communities composition and several ecosystem processes (e.g., primary and secondary production patterns), in both terrestrial and marine ecosystems^8^,^9^,^10^,^11^,^12^,^13^.

The deep sea is the largest and most remote biome of the biosphere. The deep-sea floor, covering 65% of the Earth surface, has been considered for a long time to be, with few exceptions, highly spatially homogeneous; a kind of huge and flat soft-sediment desert^14^. However, an increasing amount of information is challenging this view and expanding our understanding of the patterns and processes occurring in these ecosystems^15^,^16^. Deep-sea habitats worldwide are characterized by high spatial heterogeneity at all spatial scales: from the macro-scale, encompassing different continental margins across longitude and latitude, to the meso-scale (e.g., stable and unstable open slopes, canyons, oxygen minimum zones, cold-water coral reefs and areas of fluid seeps or venting), to the small scale (i.e., within the same habitat^4^,^5^,^6^,^17^,^18^,^19^). Also deep-sea biodiversity can vary at regional (1000 km), provincial (~100–1000 km), mega-habitat (~km-10 km) and meso-habitat (~10 m-1 km) scales^20^, with typical peaks observed at intermediate depths (i.e. around 2000 m depth^21^). At all those scales either species abundance or assemblage composition may change considerably, whereas at smaller spatial scales (<10 m) either species richness (alpha diversity) or species turnover (beta diversity) have been generally assumed to be relatively more homogenous^22^,^23^.

The discovery of highly complex and diverse communities coexisting in relatively small areas^3^,^4^,^5^,^6^,^18^,^19^,^24^,^25^,^26^,^27^ has challenged those assumptions^23^,^28^,^29^,^30^. However, to date, spatial heterogeneity in the deep sea has been mostly investigated in terms of variations in sediment type, bathymetric gradients and gradients of key environmental variables including, among the others, temperature, salinity, organic matter (OM) flux, oxygen concentration, current velocity^15^ and steadiness. There is now a large consensus that habitat heterogeneity, along with the quantity and quality of food resources represent the key factors influencing the distribution of deep-sea biodiversity, both at large and small spatial scales^4^,^5^,^6^,^15^,^31^,^32^,^33^,^34^,^35^,^36^,^37^,^38^,^39^,^40^,^41^,^42^,^43^,^44^,^45^, and the role of deep-sea temperature and its shift appears certainly important, especially over the long term^46^.

The role of deep seafloor heterogeneity on the biodiversity turnover at small spatial scales (i.e., within kilometres) is largely unknown, yet. In fact, to date, most studies on the deep sea have analyzed benthic biodiversity variations assuming each habitat as a sampling unit, relatively homogeneous from an operational point of view, for instance by contrasting canyon vs. open slope sediments but ignoring the spatial effect of the sampling stations’ position within the two habitats^47^.

Theoretical ecology predicts that heterogeneous habitats allow more species to co-exist in a given area. In the deep sea, biodiversity is most often positively and exponentially linked with ecosystem functioning^48^,^49^, so that we could expect that deep seafloor heterogeneity could influence also ecosystem functions. We also hypothesize that different seabed morphologies could be characterized by different relationships between biodiversity and ecosystem functioning.

In this study, we tested the null hypothesis by which biodiversity (using meiofaunal and nematodes as a model study), ecosystem functioning and the relationships between biodiversity and ecosystem functioning do not vary across different seafloor morphologies occurring along the slope of the South Adriatic margin (Mediterranean Sea). This area is characterized by the co-occurrence in a relatively small area of a wide variety of different seabed morphologies including: sediment waves, erosional scours and furrows^50^,^51^ (Fig. 1). More specifically, we investigated BEF relationships in eleven geo-morphological structures (a furrow, five sediment waves, a landslide scar, an erosional trough and three different other depositional structures) spread across a very limited spatial extent (average distance of <10 km between adjacent morphologies).

To test our hypothesis in the deep sea, we selected the meiofauna, the numerically dominant component of deep-sea metazoan fauna, as this component provides a good proxy of the overall deep-sea benthic biodiversity^48^. Moreover, we focused on nematodes as, among meiofauna, they typically represent >90% of total benthic faunal abundance below 200 m depth^52^,^53^, and because they can be easily assigned to different trophic guilds, allowing the analysis of functional (trophic) diversity^48^.

Since different seafloor morphologies, beside the different levels of habitat complexity, can offer different substrate characteristics and varying food availability to the meiofauna, we also explored the relationships between meiofauna (and nematode) abundance, biomass and biodiversity and sediment grain size and organic matter quantity and biochemical composition.

## Results

### Sediment features and organic components

Sediment water content, porosity, grain size, biopolymeric C and total phytopigment contents at all sampling sites are reported in Table S1. Water content ranged from 24% in the furrow to 70% in the up-slope flank of mud wave 1 and in the sediment inside the landslide scar. In the sediments of all mud waves higher water contents were reported from the up-slope flanks (53–70%) when compared with their down-slope flanks (46–50). The highest percentage of sand was recorded in the furrows field (84%), while the highest percentages of mud were recorded in mud wave and landslide scar sediments (on average 85 and 88%, respectively). Biopolymeric C contents varied from 0.36 ± 0.06 to 3.29 ± 0.41 mg C g^−1^ (in the furrow and in the landslide scar respectively, Table S1). The highest phytopigment contents occurred in the sand wave sediments (up to 25.35 ± 12.23 μg C g^−1^ in sand wave 1) and the lowest in the furrows field (1.36 ± 0.19 μgCg^−1^; Table S1).

### Faunal variables

Tables S2–S4 report the data of total meiofaunal abundance, individual nematode biomass and total meiofaunal biomass, presence/absence contingency data of meiofaunal assemblages in the different seafloor morphologies and indices of diversity. Higher values of meiofaunal abundance and biomass, and richness of higher taxa occurred in the trough (532.8 ± 192.5 ind 10 cm^−2^ and 88.8 ± 28.4 μgC 10 cm^−2^, 0.12 ± 0.07 μgC ind^−1^ and 11 higher taxa, respectively). The vertical distribution of meiofaunal abundance and biomass varied among seabed morphologies, with an accumulation in the top first cm of the sediment in all stations, with the exception of the furrow and the sediment waves (Figure S1).

Nematodes, copepods, polychaetes, kinorhynchs, tardigrades and priapulids were ubiquitous in all the investigated seafloor morphologies. Gastrotrichs, amphipods, decapods and gastropods were exclusively encountered in sediment waves, whereas oligochaetes and bryozoans were exclusive of the sediments of the trough and of the other depositional structures, respectively (Table S3).

Both the richness of meiofaunal taxa and nematode species richness were higher in sediment waves (n = 13 and SR = 176, respectively) than in all other seabed morphologies, whereas the lowest values occurred in furrow (n = 7 and SR = 52) and draped sediments (n = 6 and SR = 68). At the regional scale, 16 out of the 22 known meiofaunal taxa and a total of 250 nematodes species were found in the sediments of the study area. ANOSIM tests revealed no significant differences in the composition of meiofaunal communities and nematode assemblages among structures belonging to the same type of morphology (with the single exception for nematode species richness among two mud waves), nor among positions in selected seabed morphologies (Table S5). However, the ANOSIM tests carried out on nematode species composition (Table S5) revealed significant differences between the sediment waves and the furrow (SIMPER: 74% dissimilarity), between the sediment waves and the scar (72%), between the sediment waves and other depositional structures (70%), between the furrow and the scar (79%), between the furrow and the other depositional structures (78%), and between the scar and the trough (73%).

Nematode species richness is lower in the furrow and scar sediments than in all other morphologies (Table S4). Among the 250 identified nematode species (belonging to 160 nematode genera and 36 families; Table S6 and S7), the number of exclusive species for each seafloor morphology ranged from 10 to 45 in draped sediments and the sediment waves, respectively. *Anoplostomatidae* and *Coninckiidae* families were present exclusively in the scar, *Bodonematidae* were found only in the furrow, whereas *Lauratonematidae, Rhabdodemaniidae* and *Symplocostomatidae* were found exclusively in the sediment waves (Table S6). *Sabatieria* was the most abundant nematode genus in all the seafloor morphologies (from 17 to 28% in other depositional structures and scar, respectively), while in the draped sediments *Desmodora* was dominant (14%) followed by *Halomonhystera* (12%; Table S7). No significant differences in nematode species composition were encountered between up- and down-slope sediments of the mud waves, whereas significant differences were observed between sediments raised from inside and outside of a slide scar and inside and outside an erosional trough (Fig. 2). The number of exclusive species in each structure ranged from 2 to 13 (in the depositional structure 3 and in the sand wave 1, respectively). The number of exclusive nematode species was higher in the up-slope flank of mud waves 1 and 3 than in their down-slope flanks, and it was highest in the sediments outside the trough and the slide scar. The trophic structure of nematode assemblages in all of the investigated seafloor morphologies is dominated (range from 58 to 68%) by deposit feeders (1A and 1B; Table S4).

### Relationship between environmental variables and meiofaunal abundance, biomass and diversity

Results of the multivariate multiple regression analysis (DISTLM) are reported in Table 1, and illustrated after a distance-based redundancy analysis (dbRDA) (Fig. 3). Water depth and distance from the shore of the different seabed morphologies can explain most of the variations in the meiofaunal abundance, biomass and diversity, and the fresh primary organic matter (phytopigment content, one of the possible the main food sources for meiofauna) is responsible for a negligible fraction of variance (ca 4%; Table 1; Fig. 3a). Variations in nematode species composition were not explained by water depth and distance from the shore of the different seabed morphologies and only the 10% is significantly explained by the environmental variables available (Table 1; Fig. 3b).

### Relationship between ecosystem and functioning

Relationships between biodiversity and ecosystem functioning and efficiency across the investigated seabed morphologies are illustrated in Fig. 4. A positive exponential pattern is identified between diversity (expressed as ES(51)) and both ecosystem functioning (expressed as meiofaunal biomass) and ecosystem efficiency (expressed as the ratio between meiofaunal biomass and biopolymeric C). The Pearson’s correlation coefficient between diversity (expressed as ES(51)) and both ecosystem functioning (expressed as meiofaunal biomass) and ecosystem efficiency (expressed as the ratio between meiofaunal biomass and biopolymeric C) in the different investigated seabed morphologies is reported in Table 2. Furrow, scar and trough showed a positive relation, while no correlation was detected for other seabed morphologies.

## Discussion

### (Re)Scaling biodiversity distribution in the deep seascape

During the last decade the intense exploration of the deep ocean, along with the refinement of the available technologies has revealed the presence of a wide variety of different topographic and morphological features of the sea bed^14^, resulting in a wide variety of deep-sea habitats^54^. Among these, the most intensively investigated habitats worldwide include continental slopes and bathyal plains, as well as hydrothermal vents, and, more recently, submarine canyons and seamounts^14^. These investigations provided increasing evidence of the presence of a large number of deep seabed morphologies such as funnels, scars, troughs, mud volcanoes, and pockmarks each characterized by peculiar geological, topographic and hydrodynamic features^10^,^51^,^55^, hosting endemic species thus contributing notably to deep-sea genetic, phenotypic and functional beta diversity.

The deep sea supports a rich and highly specialised benthic fauna that varies in composition and biodiversity across multiple spatial scales^7^,^56^. Such high levels of biodiversity have been attributed to different equilibrium (linked with temporal stability) and non-equilibrium (linked to disturbance, spatial heterogeneity and dynamic forces) hypotheses^27^. In this regard, an increasing attention has been paid to address the spatial scale at which deep-sea benthic communities vary^2^,^3^,^6^,^7^,^26^, and different studies revealed the presence of significant variations also at the local and even micro scales (e.g., mud volcanoes, hydrothermal vents, coral mounds, but also single animal colonies or specimens including sponges, sea pens, xenophyophores^4^,^6^,^18^,^19^,^36^,^37^,^42^,^43^,^57^).

We focused our study in an area off the SW Adriatic Margin selected for the presence of a large variety of seabed morphologies, which determine a marked heterogeneity at relatively small spatial scales (all sea bed morphology within a distance of 10 km), which makes this region ideal to test for the effects of spatial heterogeneity on deep-sea biodiversity and ecosystem functioning, and their relationships. The high seabed heterogeneity in this region is promoted by the presence of bottom currents, which have shaped the deep-sea Adriatic seafloor (as depositional structures) and, in areas where they are particularly intense, have eroded the seafloor, leading to the formation of scours, moats or furrows^50^.

Working in the deep-sea is extremely difficult for several reasons, including the high costs related to the use of infrastructures (ships, ROV and sophisticated equipment) and the difficulty in obtaining a highly replicated number of samples. These problems are even more evident when, as in the case of the present study, replicate samples are needed from specific habitat and seafloor morphologies of limited spatial extent. Despite these intrinsic limits, which hamper generalizations, we report here that meiofaunal abundance and biomass varied among the different seafloor morphologies investigated, whereas no significant differences were observed within the single seafloor morphology.

The very low meiofaunal abundances observed in the furrow along the SW Adriatic margin, moulded by bottom currents, are analogues of deep-sea sites exposed to strong currents in the north east Atlantic^58^,^59^, and suggest that the hydrological regime can influence deep-sea assemblages. Thus different seafloor morphologies determining the presence of different environmental conditions can influence meiofaunal abundance and biomass across habitat types. In this regard, previous studies pointed out that structural sediment characteristics (i.e., heterogeneity in grain size composition) provide limited information for understanding meiofaunal distribution at different horizontal spatial scales and in different habitats^34^,^60^. Accordingly, we also report here that none of the other environmental variable considered (including the availability of resources), explained a significant proportion of meiofaunal abundance and biomass variations among different morphologies (Table 1). The difference between the present and previous studies, which reported the importance of food resources for meiofaunal distribution^32^,^33^, is likely due to the spatial scale considered (i.e., comparison between Mediterranean basins), and thus to the different primary productivity and organic matter export to the deep sea, which is not the case of the area investigated here, characterised by homogeneous levels of productivity.

Also the vertical distribution of meiofaunal abundance and biomass varied according to seafloor morphologies, with the presence of specific patterns in furrows and in sediment waves (Figure S1). These results confirm the importance of abiotic constraints acting at the centimetre scale on the vertical distribution of meiofaunal abundance and biomass in deep-sea sediments^6^.

All of these results suggest that the importance of environmental variables in influencing meiofaunal abundance and biomass distribution depends also on the spatial scale considered. Moreover, our results suggest that different factors play a prominent role in control of meiofaunal variables at increasing spatial scales of observation (i.e., from the cm scale to the meso-scales). At the microscale, the distribution of meiofaunal variables is controlled by the physical-chemical characteristics of the sediment, whereas the seabed morphology, at larger spatial scales, and the availability of resources (i.e. productivity)^7^,^35^, at basin scale play also relevant roles.

### The role of seafloor heterogeneity on meiofaunal biodiversity

We show here that also meiofaunal taxa and nematode species richness vary among different seafloor morphologies, but not among different sampling units belonging to the same seafloor morphology nor among different positions within a single topographic structure. Furrows, scars, sand and draped sediments showed lower values of biodiversity (on average from 20 to 40% in terms of meiofaunal taxa and nematode species) than all other seafloor morphologies. Firstly, this result suggests that the environmental factors that discriminate different seabed morphologies (including the distance from the coast/water depth) can influence in similar ways the α biodiversity both at species level and at the level of higher taxa. Moreover, our results also suggest that the environmental conditions characterizing clusters of different seabed morphologies are relatively invariant, so that richness of higher taxa, likewise nematode species richness, do not vary among different positions within each cluster (i.e., within each seabed morphology), whereas they vary considerably among different seabed morphologies.

Nematode species richness is influenced by a variety of processes, only in part operating across spatial scales^5^,^6^,^61^. The turnover of nematode species among seabed morphologies (Table S5) is similar with those observed in previous studies at much larger spatial scales (e.g., among different deep-sea slopes)^62^. The seafloor structures investigated here were dominated by *Comesomatidae, Chromadoridae, Sphaerolaimidae* and *Desmodoridae*, previously reported in deep-sea sediments of the South-Western Adriatic Margin^62^. Among the 250 nematode species identified only one was ubiquitous to all sampling sites (*Sabatieria sp1*). We also observed that *Metasphaerolaimus sp2* and *Wieseria sp3* were encountered only in the sand waves, while *Pareurystomina sp1* and *Platycoma sp1* were found exclusively in the sediments inside and outside the trough. Twenty-five species were exclusively found in mud wave sediments, 20 in the sand waves, 15 in the trough and in the other depositional structures and 4 in the scar. These results suggest that in the region under scrutiny the presence of different seabed morphology have allowed the establishment of highly diversified nematode communities, even in a relatively small area.

Recent developments of meta-community theory have investigated the interplay between regional and local processes in communities at the mesoscale^63^,^64^. Environmental heterogeneity and propagules dispersal between sites are two key processes behind meta-community theory^63^ and they have the potential to affect local community composition^65^,^66^,^67^,^68^. In this study, different nematode assemblages were identified by the canonical analysis of principal coordinates, where three seafloor morphologies (trough, scar and mud waves) clustered separately (Fig. 2). Overall, variations in nematode species composition among seafloor morphologies were significantly explained by only the 10% of environmental variables (Table 1). Nevertheless, nematode assemblages were indeed influenced by different sediment sorting, location and hydrodynamic conditions. The presence of strong bottom currents typical of the investigated area could contribute to the dispersal of the nematodes^69^ thus promoting a partial homogenization of communities over broad scales. Consequently, on the one hand, the lack of significant variations in the composition of meiofaunal assemblages among different seafloor morphologies could be due to the ecology of different orders to phyla present, which often show cosmopolitism^69^, but on the other hand, the large species turnover of nematode assemblages among seafloor morphologies is primarily explained by the environmental heterogeneity. This result suggests that different seafloor morphologies are colonised by different deep-sea nematode assemblages, depending on specific biogeochemical and environmental characteristics of the sediments^4^,^5^,^6^,^18^,^19^,^45^. Our study ultimately supports the hypothesis that, due to the multiplicative effects of beta diversity over a wide range of habitats, the higher the spatial heterogeneity of a region the higher the gamma diversity (regional diversity).

### Biodiversity-ecosystem functioning (BEF) relationships and deep seafloor heterogeneity

A large consensus about the importance of biodiversity in regulating the ecosystem functions that are responsible for the production of natural goods and services has been achieved and consolidated from both field and theoretical studies^70^,^71^, including the deep sea^72^. Previous experimental works, field studies and meta-analyses have shown that species diversity has most often a positive effect on ecosystem processes^73^,^74^,^75^,^76^,^77^. In marine ecosystems, the relationships between biodiversity and ecosystem functioning have been generally carried out using macro-fauna or macro-algae as proxies of biodiversity^77^. More recently, meiofauna, and in particular nematodes, have been utilized for investigating, though only with a correlative approach, the linkages between biodiversity and functioning in marine sediments^48^,^78^. The positive and exponential trend of the relationship between biodiversity and ecosystem functioning and efficiency reported in the present study (Fig. 4), have now solid confirmations from either field works on deep-sea ecosystems^48^,^49^ and theoretical analyses^79^. At the same time, when the results are analysed separately for each seafloor structure/morphology, our results indicate that in some cases the high biodiversity in the trough (blue circles in Fig. 4) are associated to higher rates of ecosystem processes, whereas in other depositional structures these effects are less evident. Thus, although the exponential patterns of BEF relationships with increasing biodiversity characterize the investigated region, specific deep-sea morphologies can shape the biodiversity-ecosystem functioning relationships, by selecting the species responsible for the effect on ecosystem processes. In previous terrestrial and marine studies, primary productivity is the most common measure of ecosystem functioning^73^,^80^. Since the deep sea lacks primary productivity, benthic biomass or change in biomass have been most often used as alternatives^48^,^49^. Our choice of using meiofaunal biomass as a proxy for deep-sea ecosystem functioning is supported by the dominance of meiofaunal biomass over macro- and megafauna biomass below 1000 m depth^24^. Nevertheless, we must acknowledge that other descriptors of ecosystem functioning could be also taken into account, including, for instance prokaryotic production or organic matter degradation rates^48^. We therefore anticipate that further studies including different types of independent ecosystem functions are needed to better understanding the linkages between BEF relationships and deep seafloor heterogeneity.

Seafloor integrity and its heterogeneity are important factors for maintaining a high deep-sea biodiversity^14^. Recent studies have shown that deep-sea floor can be severely modified by trawling for fisheries, or deep-sea mining and by drilling for oil^81^,^82^,^83^. The impact on seamounts and deep-water corals is already evident in several deep-sea regions^84^. Our results, for the first time, provide evidence that the presence of a heterogeneous seafloor characterised by different morphologies contribute to sustain high levels of deep-sea biodiversity. These, in turn, at least in the investigated deep-sea region, promote high levels of ecosystem functioning. The results of the present study need confirmations from a wider set of seafloors and biogeographic regions, in order to understand if the results reported here are specific of highly oligotrophic regions, such as the deep Mediterranean Sea, or have wider valence. Nonetheless, on the light of our results we conclude that the heterogeneous seafloors of the deep sea (including both biostructures^85^ and non-biogenic structures) should be protected in order to preserve their biodiversity and services^86^, linked to their functioning.

## Methods

### The study area and sampling strategy

**Figure 1:**
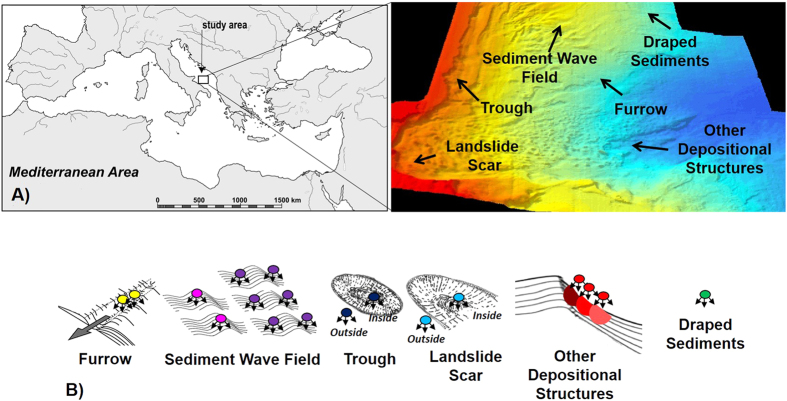
Study area and sampling sites. (**A**) Map of the sampling sites in the South-Western Adriatic Margin (Central Mediterranean Sea) and digital elaboration of seafloor geomorphology obtained by side-scan sonar and multi-beam data modified from Trincardi *et al*.^51^. Details about multi-beam and side-scan sonar methods are reported in Supplementary Information session. Schematic representation of sampling sites in seafloor morphologies (**B**): furrow 1a and 1b in yellow, sand waves 1 and 2 in pink, mud waves 1, 2 and 3 in violet, inside and outside the trough in blue, inside and outside the scar in light blue, other depositional structures 1, 2 and 3 in red, draped sediments in green.

**Figure 2:**
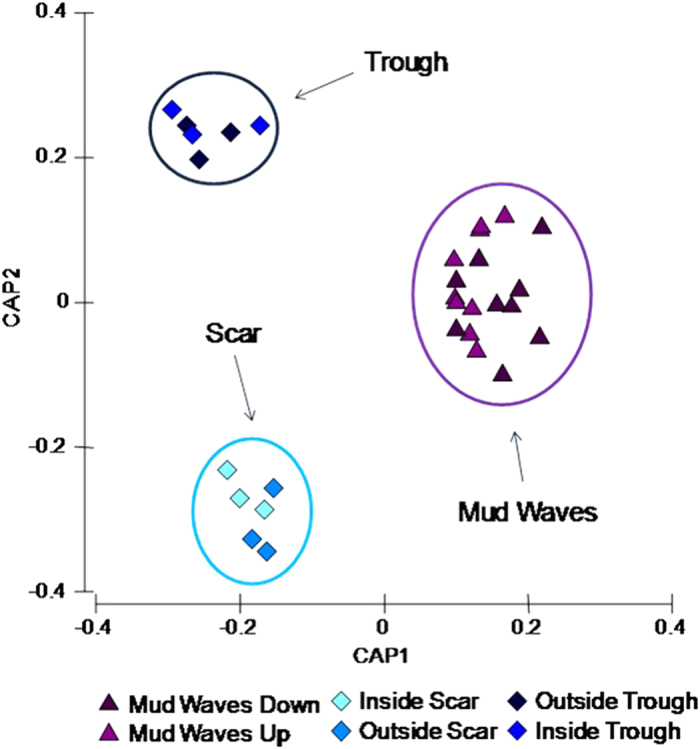
Comparison of mud waves, scar and trough. Reported are results of the canonical analysis of principal coordinates based on nematode species composition. Data were presence/absence transformed.

**Figure 3:**
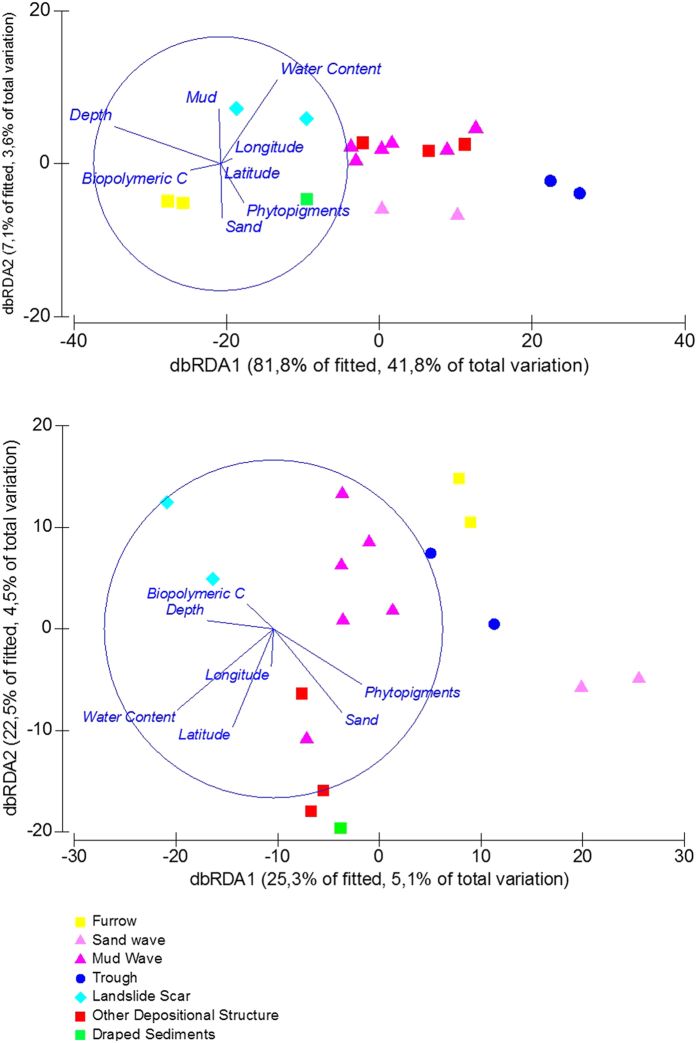
Relationships between the environmental variables and meiofauna/nematode community. Distance-based redundancy analysis ordinations to investigate relationships between the environmental variables and meiofauna community (**A**) and nematode community (**B**).

**Figure 4:**
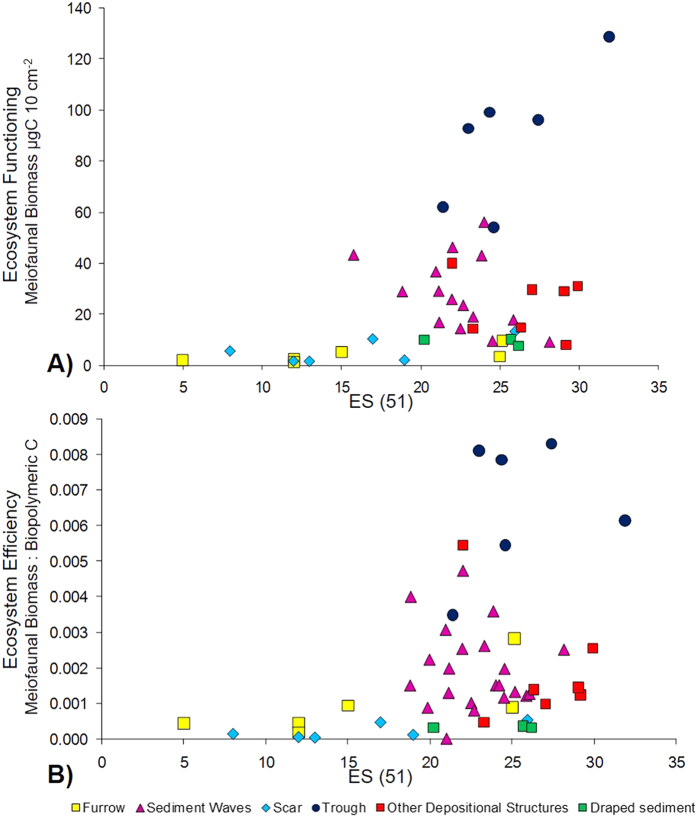
Relationship between biodiversity, ecosystem functioning and efficiency. The relationship between expected species number (ES(51)) and ecosystem functioning (as faunal biomass, expressed as μg C 10 cm^−2^, (**A**)). The relationship between expected species number (ES(51)) and ecosystem efficiency as the ratio of faunal biomass to biopolymeric (**C**) (as a measure of the bioavailable organic detritus, (**B**)).

The south-western Adriatic Margin (Fig. 1A) is markedly steep and erosional above ca. 350 m water depth but more in depth, where the slope is not erosional, a large variety of bed forms and sediment drift deposits are present^5^,^50^,^51^,^59^. In this area, after a survey of sea bottom profiling (Supplementary Information), we identified 11 seabed morphologies characterised by 7 different topographies (i.e., Fig. 1B): 1) furrows (n = 1), 2) sand waves (n = 2), 3) mud waves (n = 3), 4) landslide scar (n = 1), 5) other depositional structures (N = 3), 6) trough (n = 1) and 7) draped sediments (n = 1), where no erosional or depositional structures were recognized. Samples were collected either inside and outside the landslide scar and the trough, and along two opposite flanks (up-slope and down-slope) for each of the 3 mud waves. The exact location, the description of the areas and the depth of all investigated areas are reported in Table S1.

Sediment samples were collected on board the R/V Urania during the cruise SETE_06 (sand wave 1 and 2; May 2006) and the cruise BARCA_07 (all other areas; March-April 2007). At each area, undisturbed sediment samples were collected using a NIOZ-type box-corer, with three independent deployments of the box-corer per area. From each box-corer sediment samples were collected using Plexiglas corer (area 19.6 cm^2^, 20 cm depth). For the analyses of the organic components, the first cm of three sediment cores from independent box-corer deployments was frozen at −20 °C until analysis (within two weeks). One sediment corer was dedicated to sediment analyses. For meiofaunal analyses, at each site, three sediment cores from independent box-corer deployments were collected and sectioned into five layers (0–1, 1–3, 3–5, 5–10, 10–15 cm). Since the analysis of the vertical distribution of meiofaunal assemblages generally showed the greatest abundance in the top first centimetre (Figure S1), the data were integrated down to 15 cm depth in the sediment. All samples for meiofaunal analyses were preserved in buffered 4% formalin solution and stained with Rose Bengal.

### Sedimentary features and analysis of the organic components

Sediment grain size analyses were carried out after treatment with 10% hydrogen peroxide for 48 h to destroy organic aggregates. The muddy fraction was separated by wet sieving through a 63-μm mesh sieve. Sand was separated from shell debris by dry sieving using a 250-μm mesh sieve. Clay and silt contents within the muddy fraction were determined using by a Micromeritics X-ray sedigraph after dispersion in sodium hexametaphosphate solution and subsequent ultrasonic disaggregation (10–15 min). Porosity (β) of sediments was calculated using the formula β  = 0.3105 + 0.0552ϕ 71, where ϕ is –log_2_*d*, and *d* is the grain diameter in mm. Water content, expressed as percentage of sediment wet weight, was calculated using the equation W = 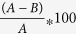, where A is the weight (g) of wet sample, and B is the weight (g) of dry sample.

Chlorophyll-a and phaeopigment sedimentary contents were determined fluorometrically according to Lorenzen and Jeffrey^87^. Pigments were extracted (12 h at 4 °C in the dark) from triplicate sediment samples (about 1 g each) using 3–5 mL of 90% acetone. To determine the phaeopigment concentrations, the fluorescence of the extracts was measured after acidification with 200 μl of 0.1 N HCl. Concentrations are reported as μg g DW^−1^. Since chlorophylls’ degradation products are typically dominant in deep-sea sediments^88^, we summed up chlorophyll-a and phaeopigment concentrations (i.e. total phytopigments) and assumed to represent the labile fraction of sedimentary organic matter^89^.

Protein, carbohydrate and lipid sedimentary contents were determined following spectrophotometric protocols^90^. For each biochemical assay, blanks were obtained using pre-combusted sediments (450 °C for 4 h). Analyses were performed in 3 replicates on about 1 g of sediment for each sediment sample. Carbohydrate, protein and lipid contents were converted into carbon equivalents using the conversion factors of 0.40, 0.49 and 0.75 μgC μg^−1^, respectively, their sum reported as the biopolymeric carbon and assumed to represent the semi-labile fraction of sedimentary organic matter (OM)^91^.

### Meiofaunal abundance, biodiversity and biomass

Sediments were sieved through a 1,000-μm mesh, and a 20-μm mesh was used to retain the organisms. These were re-suspended and centrifuged three times with Ludox HS40 (density 1.31 g cm^−3^). All meiofaunal animals were counted and classified under a stereomicroscope at the phylum level. Soft-bodied organisms were mounted on slides and viewed at 1,000× magnification. The abundance of the meiofauna is reported as individuals 10 cm^−2^. Meiofaunal biomass was calculated from the biovolume using the Andrassy formula (V = L × W^2^ × 0.063 × 10^−5^, in which body length, L, and width, W, are expressed in μm)^92^. Body volumes were derived from body length (L, in mm) and width (W, in mm) measurements, using the formula V = L × W^2^ × C, where C is the approximate conversion factor for each meiofaunal taxon^93^. Body volume was multiplied by an average density (1.13 gcm^−3^) to obtain the biomass (μg DW: μg WW = 0.25)^93^, and the carbon content calculated as the 40% of the dry weight^93^.

### Nematode diversity

A total of 100 nematodes (or all of the retrieved nematodes if <100) were randomly picked up from each of the three independent samples at each site and mounted on slides after formalin-ethanol-glycerol treatment. Nematodes were identified at the genus level and, whenever possible, at the species level, according to Platt and Warwick^94^,^95^, Warwick *et al*.^96^ and the recent literature (NeMys database^97^). Unknown species were reported as *Genus* sp1, sp2 etc.

Nematode diversity was measured by Shannon-Wiener diversity index (H’, using log_e_) and evenness as J^98^. Species Richness (SR) was calculated as the total number of species identified in each station. The Margalef diversity index (D) was estimated as D = (S − 1)/lnN, where S is the number of species and N is the number of individuals in the sample. The Simpsons diversity index (SI = 1 − ∑(Pi)^2^, where Pi = percent of species “ι“ in the total abundance). For each site, the expected number of species for a theoretical sample of 51 specimens, ES(51), was calculated^99^.

Nematodes were assigned each to one of the four following groups: (1A) no buccal cavity or a fine tubular one -selective (bacterial) feeders; (1B) large but unarmed buccal cavity-non-selective deposit feeders; (2A) buccal-cavity with scraping tooth or teeth-epistrate or epigrowth (diatom) feeders; (2B) buccal cavity with large jaws-predators/omnivores^100^. The functional diversity of nematodes was estimated as 1-Θ, with Θ = g_1_^2^ + g_2_^2^ + g_3_^2^ + g_4_^2^, and g_n_ is the relative contribution of each of the four identified trophic groups to the total number of individuals^101^. 1-Θ ranges from 0.75 (highest functional diversity; i.e. the four trophic guilds account each for 25% of total nematode abundance) to 0.25 (lowest functional diversity; i.e., one trophic guild accounts for 100% of total nematode abundance).

### Ecosystem functioning and efficiency

Five groups of ecosystem functions were proposed by Strong *et al*.^102^: biomass, organic matter transformation, ecosystem metabolism, nutrient cycling and physical engineering. In this study ecosystem functioning was measured in terms of total meiofaunal biomass and ecosystem efficiency calculated as the ratio of benthic faunal biomass and food supply (here quantified as biopolymeric C contents^91^).

### Statistical analyses

Mean, sorting, skewness and kurtosis sediment statistics were calculated arithmetically and geometrically (in metric units) and logarithmically (in phi units) using moment and Folk and Ward graphical methods, under the GRADISTAT v 4.0 software.

ANOSIM and SIMPER tests were carried out to measure the similarities in the nematode and higher taxa compositions, separately, between different seafloor morphologies (furrow, sediment waves, scar, trough, other depositional structures and draped soft sediments) and between distinct physical structures belonging to a single type of morphology (i.e. comparing each other three depositional structures, three mud waves and two sand waves). Variations in the composition of meiofauna and nematode communities were estimated as the percentage of dissimilarity, based on a Bray-Curtis similarity matrix^103^. The analysis of dissimilarities was based on a presence-absence matrix (SIMPER analysis). SIMPER decomposes average Bray-Curtis dissimilarities between all pairs of samples, one from each group (or decomposes all similarities among samples within a group) into percentage contributions from each species, listing the species in decreasing order of such contributions^104^. γ diversity was calculated as the total species number of the investigated region.

To evaluate the fraction of variance in meiofaunal abundance, biomass, taxa and species richness, and meiofaunal phylum and nematode species compositions explained by environmental covariates a non-parametric multivariate multiple regression analysis was carried out^105^. Environmental covariates included latitude, longitude, water depth, percentage of mud and sand in the sediment, and water, phytopigment and biopolymeric C sedimentary contents. For the meiofauna and nematode assemblage composition, regressions were carried out using Bray–Curtis similarity matrixes based on square root and presence/absence transformed data, respectively. Distance-based redundancy analysis (dbRDA) was applied to visualize relationships between the environmental variable and meiofauna/nematode assemblages. All statistical tests were carried out using the dedicated routines included in the PRIMER6+ software. Pearson Correlation Coefficient was calculated for the correlation between diversity and ecosystem functioning (Excel, version 2013).

## Additional Information

**How to cite this article**: Zeppilli, D. *et al*. Seafloor heterogeneity influences the biodiversity–ecosystem functioning relationships in the deep sea. *Sci. Rep.* **6**, 26352; doi: 10.1038/srep26352 (2016).

## Supplementary Material

Supplementary Information

## Figures and Tables

**Table 1:** Results of the multivariate multiple regression analysis carried out on the meiofaunal abundance and biomass, richness of higher taxa, nematode species richness and meiofaunal higher taxa and nematode species composition between all investigated sites.

	Variable	F	P	% Var	% Cum
Meiofaunal Abundance and Biomass	Depth	55.29	***	52	52
	Longitude	7.32	*	6	58
	Phytopigments	5.04	*	4	62
	Biopolymeric C	2.42	ns	2	63
	Latitude	1.73	ns	1	65
	Mud%	0.84	ns	1	65
Higher Taxa Richness	Depth	14.72	***	22	22
	Water Content	14.72	***	17	40
	Longitude	0.93	ns	1	41
	Biopolymeric C	0.69	ns	1	41
	Mud%	0.17	ns	0	42
Nematode Species Richness	Phytopigments	2.41	ns	4	4
	Depth	1.55	ns	3	7
	Mud%	1.48	ns	3	10
	Water Content	0.65	ns	1	11
	Biopolymeric C	0.39	ns	1	12
Meiofaunal Higher Taxa Composition	Depth	21.94	***	30	30
	Longitude	7.29	***	9	38
	Phytopigments	3.69	**	4	43
	Water Content	2.57	*	3	46
	Sand%	1.24	ns	1	47
	Biopolymeric C	1.64	ns	2	49
Nematode Species Composition	Water Content	1.89	**	4	4
	Sand%	1.91	**	3	7
	Phytopigments	1.68	*	3	10
	Depth	1.30	ns	2	12
	Longitude	1.14	ns	2	14

% Var = percentage of explained variance (***p < 0.001; **p < 0.01; *p < 0.05; ns not significant).

**Table 2:** Pearson’s Correlation Coefficient between meiofaunal biomass expected species number (ES(51)) and ecosystem functioning (as faunal biomass, expressed as μg C 10 cm^−2^) and between expected species number (ES(51)) and ecosystem efficiency as the ratio of faunal biomass to biopolymeric C (as a measure of the bioavailable organic detritus) in the different investigated seabed morphologies.

	ES(51)/Biomass	ES(51)/Ecosystem Efficiency
Furrow	0.683100824	0.714600032
Sediment Waves	−0.276951183	−0.122297939
Scar	0.624437333	0.714083482
Trough	0.766890811	0.252198204
Other Depositional Structures	−0.200291751	−0.407628791
Draped Sediments	−0.508599959	0.622419057
