# Supplementary material for: Seafloor heterogeneity influences the biodiversity–ecosystem functioning relationships in the deep sea
Source: Sci Rep. 2016 May 23;6:26352. doi: 10.1038/srep26352 (PMC4876447; doi:10.1038/srep26352)

Seafloor heterogeneity influences the biodiversity–ecosystem functioning relationships in the deep sea

Daniela Zeppilli[1,2], Antonio Pusceddu[1], Fabio Trincardi[3], Roberto Danovaro[1,4]

[1]*Department of Life and Environmental Sciences, Università Politecnica delle Marche, Via Brecce Bianche, 60131 Ancona, Italy*

[2]*IFREMER, Centre Brest, REM/EEP/LEP, Institut Carnot Ifremer-EDROME, ZI de la pointe du diable, CS10070, F-29280 Plouzané, France*

[3]*ISMAR (CNR), via Gobetti 101, 40129 Bologna, Italy*

[4]*Stazione Zoologica Anton Dohrn, Villa Comunale, 80121, Naples, Italy.*

Supplementary Information

Seabed digital mapping

A bathymetric map of the South-Western Adriatic Margin was acquired using a Kongsberg-Simrad EM300 multi-beam echo 125 sounder with nominal sonar frequency of 30 kHz and angular coverage sector of 135 beams per 126 ping at 1°. The multibeam data processing was carried out using CARIS HIPS and SIPS 7.0. The processing methodology applied implies the creation of a 2D and 3D best-fit interpolated surface using different algorithms and grid resolutions. Following the analysis of the data errors, the definition of a strategy to solve them includes: 1) the correction of the motion sensor calibration parameters; 2) the sound speed correction applying a ray-tracing technique; 3) the manual cleaning of the spikes (beam removal) in the areas where they are visible on the 2D and 3D surface; 4) the automatic filtering for a depth window, by beam number or slope between points. After the data correction and cleaning, the surface obtained is converted in ASCII ESRI format and merged with the mosaic tool available in ArcGIS 9.3. The ArcGIS Spatial Analyst tool was applied to derive the hill shade, the contours and the slope maps from the DTM.

Chirp-sonar profiles were gathered using a hull-mounted 16-transducer source with a sweep modulated 2-7-kHz outgoing signal equivalent to a 3.5 kHz profiler, allowing vertical resolution of 0.5 m or better. High-resolution seismic profiles were gathered using a 16 transducers hull-mounted Chirp-sonar profiler with a sweep modulated outgoing signal equivalent to 3.5 kHz with vertical resolution of 0.5 m or less. Seafloor morphology and backscatter pattern come from a 30 kHz TOBI side scan sonar mosaic acquired during the cruises SETE_06 and BARCA_07. Differential GPS assured ship positioning every 5 s; TOBI was positioned by calculating accurately its distance behind the ship using a HPR.

Table S1. Location, depth, typology, water content, porosity and percentage of mud and sand in the sediments, mean, sorting, skewness and kurtosis of the grains, biopolymeric carbon and phytopigments contents in the investigated sediments (sd = standard deviation; na= not available).

**Latitude (N)**	**Longitude (E)**	**Depth (m)**	**Sampling site typology**	**Water Content (%)**	**Porosity**	**Mean (X)**	**Sorting (σ)**	**Skewness (Sk)**	**Kurtosis (K)**	**Sand (%)**	**Mud (%)**	**Biopolymeric C** mg C g^{-1}	sd	**Phytopigments** µg g^{-1}	sd
41° 47.34’	17° 01.85’	714	Furrow 1a	24	0.4	2.2	3.1	0.8	2.1	84	16	0.36	0.06	3.04	0.77
41° 47.34’	17° 01.84’	712	Furrow 1b	24	0.4	2.2	3.1	0.8	2.1	84	16	0.56	0.10	1.36	0.19
41° 52.56'	16° 56.86'	525	Sand Wave 1	43	0.5	4.2	4.2	0.5	0.6	64	36	1.55	0.92	25.35	12.23
41° 50.48'	16° 59.08'	596	Sand Wave 2	35	0.5	4.2	4.2	0.6	0.6	64	36	0.55	0.28	18.59	6.75
41° 52.54'	17° 00.47’	590	Mud Wave 1down	47	0.6	5.4	4.4	0.0	0.5	48	52	1.05	0.10	3.15	0.21
41° 52.79’	17° 00.33’	572	Mud Wave 1up	70	0.8	8.6	2.6	-0.2	0.9	8	92	1.61	0.06	14.58	3.38
41° 53.46’	16° 59.85'	556	Mud Wave 2 down	46	0.8	8.2	2.7	-0.1	1.0	9	91	1.11	0.09	3.06	0.67
41° 53.68'	16° 59.70'	557	Mud Wave 2 up	53	0.8	8.8	2.8	-0.3	1.0	12	88	1.78	0.05	9.42	1.35
41° 53.97’	16° 59.48'	550	Mud Wave 3 down	50	0.8	8.8	2.3	0.0	0.8	4	96	0.92	0.11	6.07	0.04
41° 54.21'	16° 59.31'	524	Mud Wave 3 up	58	0.8	8.6	2.9	-0.3	1.0	11	89	1.95	0.32	13.80	3.04
42° 02.75'	16° 51.32'	328	Inside Trough	50	0.6	5.5	4.3	0.1	0.6	48	52	1.54	0.38	4.32	1.02
42° 02.78'	16° 51.57'	318	Outside Trough	44	0.7	6.3	4.1	0.0	0.6	46	54	0.99	0.08	8.25	1.75
41° 51.76'	17° 03.49'	718	Inside Landslide Scar	70	0.8	8.5	2.9	-0.3	1.1	12	88	3.29	0.41	4.22	0.99
41° 51.62'	17° 02.48'	667	Outside Landslide Scar	63	0.8	8.5	2.9	-0.3	1.1	12	88	1.92	0.34	3.85	0.61
41° 39.31'	17° 08.38'	637	Depositional Structure 1	56	0.5	3.6	3.6	0.5	2.5	79	21	0.64	0.04	2.41	0.47
41° 39.47'	17 °08.14'	620	Depositional Structure 2	63	0.5	3.7	3.6	0.5	1.9	77	23	1.01	0.07	4.24	0.47
41° 40.13'	17° 08.82'	681	Depositional Structure 3	65	0.5	3.1	3.9	0.9	0.9	75	25	2.45	0.56	6.04	1.04
42° 13.95’	16° 54.22’	584	Draped sediments	38	na	na	na	na	na	71	29	2.51	0.40	8.78	1.17

Table S2. Total meiofaunal abundance, individual nematode biomass and total meiofaunal biomass (sd = standard deviation) in the sampled sites.

Sampling Site	Total meiofaunal abundance		Individual nematode biomass		Total meiofaunal biomass	
	ind 10cm^{-2}	sd	µgC ind^{-1}	sd	µgC 10cm^{-2}	sd
Furrow 1a	114.0	88.1	0.04	0.01	5.1	4.0
Furrow 1b	53.6	12.3	0.03	0.01	3.1	2.0
Sand Wave 1	280.9	88.7	0.05	0.01	29.6	21.6
Sand Wave 2	162.0	79.7	0.06	0.02	23.5	19.5
Mud Wave 1 down	180.3	77.0	0.04	0.00	14.8	4.5
Mud Wave 1 up	436.4	149.7	0.09	0.05	48.4	6.8
Mud Wave 2 down	182.0	116.3	0.15	0.08	29.4	15.9
Mud Wave 2 up	213.0	68.7	0.1	0.08	57.1	36.9
Mud Wave 3 down	206.1	115.4	0.07	0.04	24.0	13.8
Mud Wave 3 up	304.5	89.1	0.06	0.03	23.9	5.0
Inside Trough	561.2	164.3	0.12	0.06	96.6	33.4
Outside Trough	504.4	220.7	0.12	0.08	81.0	23.4
Inside Scar	47.0	7.8	0.05	0.05	2.9	2.3
Outside Scar	149.9	18.9	0.03	0.02	8.5	5.8
Depositional Struct. 1	172.2	40.5	0.13	0.09	24.4	16.0
Depositional Struct. 2	276.7	117.5	0.06	0.02	22.1	8.2
Depositional Struct. 3	290.1	72.3	0.05	0.02	24.4	8.6
Draped Sediments	126.7	35.8	0.06	0.02	9.3	1.4

Table S3. Meiofaunal community structure. Distribution of higher taxa in the investigated morphologies (including draped sediments), structures, in the up-/down-slope flanks of the mud waves and in the sediment inside and outside the trough and the scar (+ indicate the presence of taxa, - indicate the absence).

	Morphologies					
	**Furrow**	**Sed. Waves**	**Trough**	**Scar**	**Dep. Structures**	**Draped Sed.**
Nematodes	+	+	+	+	+	+
Copepods	+	+	+	+	+	+
Polychaetes	+	+	+	+	+	+
Ostracods	-	+	+	+	+	-
Kinorhynchs	+	+	+	+	+	+
Oligochaetes	-	-	+	-	-	-
Tardigrades	+	+	+	+	+	+
Gastrotrichs	-	+	-	-	-	-
Amphipods	-	+	-	-	-	-
Isopods	-	+	+	+	+	-
Acarines	+	+	+	-	+	-
Priapulids	+	+	+	+	+	+
Decapods	-	+	-	-	-	-
Gastropods	-	+	-	-	-	-
Bryozoans	-	-	-	-	+	-

Table S3 (continue)

	Structures							
	**Sand Wave 1**	**Sand Wave 2**	**Mud Wave 1**	**Mud Wave 2**	**Mud Wave 3**	**Dep. Struct. 1**	**Dep. Struct. 2**	**Dep. Struct. 3**
Nematodes	+	+	+	+	+	+	+	+
Copepods	+	+	+	+	+	+	+	+
Polychaetes	+	+	+	+	+	+	-	+
Ostracods	+	+	+	-	-	+	+	-
Kinorhynchs	+	-	+	+	+	+	+	+
Oligochaetes	-	-	-	-	-	-	-	-
Tardigrades	-	-	+	+	+	+	+	+
Gastrotrichs	-	-	-	+	+	-	-	-
Amphipods	-	-	-	+	-	-	-	-
Isopods	-	-	+	-	+	-	-	+
Acarines	-	-	+	-	-	+	+	+
Priapulids	-	+	+	+	+	+	+	-
Decapods	-	-	+	-	-	-	-	-
Gastropods	-	-	-	-	+	-	-	-
Bryozoans	-	-	-	-	-	-	-	+

Table S3 (continue)

	Inside Structures									
	**Mud Wave 1**		**Mud Wave 2**		**Mud Wave 3**		**Trough**		**Scar**	
	**Down**	**Up**	**Down**	**Up**	**Down**	**Up**	**Inside**	**Outside**	**Inside**	**Outside**
Nematodes	+	+	+	+	+	+	+	+	+	+
Copepods	+	+	+	+	+	+	+	+	+	+
Polychaetes	+	+	+	+	+	+	+	+	-	+
Ostracods	+	+	-	-	-	-	+	+	-	+
Kinorhynchs	+	+	+	+	-	+	-	+	+	+
Oligochaetes	-	-	-	-	-	-	+	-	-	-
Tardigrades	+	+	+	+	+	+	+	-	+	+
Gastrotrichs	-	-	+	-	+	+	-	-	-	-
Amphipods	-	-	-	+	-	-	-	-	-	-
Isopods	-	+	-	-	-	+	+	+	-	+
Acarines	+	-	-	-	-	-	+	+	+	-
Priapulids	-	+	-	+	+	+	+	+	-	-
Decapods	-	+	-	-	-	-	-	-	-	-
Gastropods	-	-	-	-	-	+	-	-	-	-
Bryozoans	-	-	-	-	-	-	-	-	-	-

Table S4. Indices of meiofaunal and nematode diversity at all sampling sites. Reported are: richness of higher meiofauna l taxa (HTR), and, for nematodes, the Species Richness (SR), the index of Margalef (D), the Pielou's J, the expected species number ES(51), the Shannon-Wiener index (H') and the index of trophic diversity ((1–Θ); (avg = average; sd = standard deviation).

**Sampling site**	**HTR**	**SR**	**D**		**J**		**H**		**SI**		**ES(51)**		**1-Θ**	
			avg	sd	avg	sd	avg	sd	avg	sd	avg	sd	avg	sd
Furrow 1a	5	49	5.44	1.97	0.95	0.04	2.63	0.68	0.97	0.02	18.38	8.92	0.70	0.03
Furrow 1b	5	30	3.81	0.26	0.92	0.02	2.36	0.15	0.92	0.02	13.00	1.33	0.63	0.01
Sand Wave 1	5	69	6.65	0.62	0.87	0.05	2.94	0.26	0.92	0.04	23.84	1.79	0.63	0.06
Sand Wave 2	5	57	6.32	0.42	0.90	0.02	2.93	0.08	0.94	0.01	23.28	0.85	0.68	0.05
Mud Wave 1down	7	60	7.01	0.19	0.86	0.03	3.00	0.14	0.92	0.03	24.25	1.20	0.62	0.06
Mud Wave 1up	9	68	7.07	0.75	0.84	0.06	2.94	0.30	0.90	0.04	23.68	2.97	0.61	0.08
Mud Wave 2 down	6	42	5.43	0.94	0.72	0.12	1.54	1.03	0.79	0.13	12.62	7.75	0.55	0.06
Mud Wave 2 up	7	47	5.68	0.11	0.85	0.07	2.73	0.14	0.91	0.04	19.92	0.75	0.66	0.03
Mud Wave 3 down	6	49	6.00	0.25	0.86	0.03	2.84	0.10	0.92	0.01	21.60	1.85	0.58	0.07
Mud Wave 3 up	9	69	6.80	0.48	0.84	0.08	2.92	0.35	0.90	0.08	23.17	2.21	0.61	0.05
Inside Trough	10	60	6.72	1.08	0.88	0.04	2.95	0.26	0.92	0.03	25.88	4.00	0.63	0.08
Outside Trough	8	61	6.82	0.84	0.88	0.04	2.95	0.08	0.93	0.02	25.00	1.60	0.64	0.05
Inside Scar	5	24	3.30	0.57	0.91	0.04	2.17	0.17	0.91	0.03	11.00	2.00	0.54	0.06
Outside Scar	7	38	5.36	1.15	0.91	0.01	2.79	0.22	0.94	0.01	20.66	3.54	0.67	0.05
Depositional Structure 1	8	49	5.64	1.21	0.94	0.04	2.75	0.30	0.96	0.02	20.39	6.93	0.66	0.04
Depositional Structure 2	7	78	8.20	0.57	0.93	0.01	3.34	0.08	0.97	0.01	29.45	2.07	0.68	0.08
Depositional Structure 3	8	58	6.80	0.59	0.94	0.01	3.18	0.07	0.96	0.00	26.45	2.11	0.64	0.02
Draped Sediments	6	68	6.69	0.70	0.85	0.10	2.91	0.44	0.89	0.09	24.02	2.54	0.64	0.04
Total (gamma)	15	250	-	-	-	-	-	-	-	-	-	-	-	-

Table S5. Dissimilarity in meiofaunal higher taxa (HT) and nematode species compositions in the investigated morphologies and structures. Reported are the results of the SIMPER and ANOSIM tests (*** = p<0.001; ** = p<0.01; * = p<0.05; ns = not significant).

	Meiofaunal HT composition			Nematode species composition		
	ANOSIM		SIMPER dissimilarity	ANOSIM		SIMPER dissimilarity
**Contrast**	R	P	%	R	P	%
Sediment Waves vs Furrow	0.00	ns	28	0.32	*	74
Sediment Waves vs Draped Sediments	-0.14	ns	25	0.18	ns	68
Sediment Waves vs Scar	0.13	ns	32	0.31	*	72
Sediment Waves vs Trough	0.27	*	35	0.21	ns	69
Sediment Waves vs Dep. Structures	-0.06	ns	26	0.22	*	70
Furrow vs Draped Sediments	0.00	ns	23	0.08	ns	79
Furrow vs Scar	0.32	*	33	0.22	*	79
Furrow vs Trough	0.57	**	32	0.03	ns	74
Furrow vs Dep. Structures	0.08	ns	24	0.26	*	78
Draped Sediments vs Scar	-0.02	ns	26	0.15	ns	71
Draped Sediments vs Trough	0.99	*	38	0.12	ns	71
Draped Sediments vs Dep. Structures	0.21	ns	25	-0.12	ns	68
Scar vs Trough	0.89	**	48	0.35	*	73
Scar vs Dep. Structures	0.39	**	32	0.11	ns	72
Trough vs Dep. Structures	0.57	***	29	0.08	ns	71
Mud vs sand	0.17	ns	26	0.41	*	70
Mud wave 1 vs 2	0.16	ns	25	0.10	ns	66
Mud wave 1 vs 3	0.00	ns	23	0.22	*	59
Mud wave 2 vs 3	-0.11	ns	21	-0.03	ns	63
Sand wave 1 vs 2	0.07	ns	24	-0.19	ns	68
Depositional Structure 1 vs 2	0.33	ns	24	0.22	ns	74
Depositional Structure 1 vs 3	0.67	ns	27	0.30	ns	73
Depositional Structure 2 vs 3	0.22	ns	19	0.11	ns	64

Table S6. Nematode families in the investigated morphologies, structures and in the up-/down-slope flanks of the mud waves and in the sediment inside and outside the trough and the scar (+ indicate the presence of the family, - indicate its absence).

	Morphologies					
Family	Furrow	Sediment Waves	Trough	Scar	Dep. Structures	Draped Sed.
Aegialoalaimidae	+	+	+	+	+	+
Anoplostomatidae	-	-	-	+	-	-
Bodonematidae	+	-	-	-	-	-
Ceramonematidae	+	+	+	+	+	+
Chromadoridae	+	+	+	+	+	+
Comesomatidae	+	+	+	+	+	+
Coninckiidae	-	-	-	+	-	-
Cyatholaimidae	+	+	+	+	+	+
Desmodoridae	+	+	+	+	+	+
Desmoscolecidae	+	+	+	+	+	+
Diplopeltidae	-	+	+	-	-	-
Draconematidae	-	-	-	-	-	-
Enchelidiidae	+	+	+	+	+	+
Epsilonematidae	-	-	+	-	+	+
Ironidae	+	+	+	+	+	+
Lauratonematidae	-	+	-	-	-	-
Leptolaimidae	-	+	+	-	+	+
Leptosomatidae	+	+	+	-	+	+
Linhomoeidae	+	+	+	+	+	+
Meyliidae	+	+	+	+	+	+
Microlaimidae	+	+	+	-	+	+
Monhysteridae	+	+	+	+	+	+
Oncholaimidae	-	+	+	+	+	+
Oxystominidae	+	+	+	+	+	+
Pandolaimidae	+	-	+	-	-	-
Paramicrolaimidae	-	-	-	-	-	-
Phanodermatidae	+	+	+	-	+	+
Rhabdodemaniidae	-	+	-	-	-	-
Selachinematidae	+	+	+	+	+	+
Siphonolaimidae	-	+	-	-	+	+
Sphaerolaimidae	+	+	+	+	+	+
Symplocostomatidae	-	+	-	-	-	-
Thoracostomopsidae	+	+	-	-	-	-
Tripyloididae	-	+	+	-	-	-
Xyalidae	+	+	+	+	+	+

Table S6 (continue)

					Structures			
**Family**	**Sand Wave 1**	**Sand Wave 2**	**Mud Wave 1**	**Mud Wave 2**	**Mud Wave 3**	**Dep. Structure 1**	**Dep. Structure 2**	**Dep. Structure 3**
Aegialoalaimidae	+	+	+	+	+	+	+	-
Anoplostomatidae	-	-	-	-	-	-	-	-
Bodonematidae	-	-	-	-	-	-	-	-
Ceramonematidae	+	-	+	+	+	+	+	+
Chromadoridae	+	+	+	+	+	+	+	+
Comesomatidae	+	+	+	+	+	+	+	+
Coninckiidae	-	-	-	-	-	-	-	-
Cyatholaimidae	+	+	+	+	+	+	+	+
Desmodoridae	+	+	+	+	+	+	+	+
Desmoscolecidae	+	+	+	+	+	+	+	+
Diplopeltidae	-	-	+	+	+	-	-	-
Draconematidae	-	-	-	-	-	-	-	-
Enchelidiidae	+	-	+	+	+	-	+	+
Epsilonematidae	-	-	-	-	-	-	-	+
Ironidae	+	+	+	+	+	+	+	+
Lauratonematidae	-	-	+	-	-	-	-	-
Leptolaimidae	+	-	+	-	+	-	+	-
Leptosomatidae	+	-	+	-	-	+	-	+
Linhomoeidae	+	-	-	+	+	-	+	-
Meyliidae	+	+	+	+	+	+	+	+
Microlaimidae	+	+	+	+	+	+	+	+
Monhysteridae	+	+	+	+	+	+	+	+
Oncholaimidae	-	+	+	+	+	-	+	-
Oxystominidae	+	+	+	+	+	+	+	+
Pandolaimidae	-	-	-	-	-	-	-	-
Paramicrolaimidae	-	-	-	-	-	-	-	-
Phanodermatidae	+	+	+	+	+	-	+	+
Rhabdodemaniidae	-	+	-	-	-	-	-	-
Selachinematidae	+	+	+	+	+	+	+	+
Siphonolaimidae	-	+	-	-	-	-	+	-
Sphaerolaimidae	+	+	+	+	+	+	+	+
Symplocostomatidae	-	-	-	-	+	-	-	-
Thoracostomopsidae	-	-	+	+	+	-	-	-
Tripyloididae	+	-	-	+	+	-	-	-
Xyalidae	+	+	+	+	+	+	+	+

Table S6 (continue).

	Inside Structures									
	**Mud Wave 1**		**Mud Wave 2**		**Mud Wave 3**		**Scar**		**Trough**	
**Family**	**Down**	**Up**	**Down**	**Up**	**Down**	**Up**	**Inside**	**Outside**	**Inside**	**Outside**
Aegialoalaimidae	-	+	-	-	+	+	-	+	+	-
Anoplostomatidae	-	-	-	-	-	-	-	+	-	-
Bodonematidae	-	-	-	-	-	-	-	-	-	-
Ceramonematidae	+	+	+	+	+	+	+	+	+	+
Chromadoridae	+	+	+	+	+	+	+	+	+	+
Comesomatidae	+	+	+	+	+	+	+	+	+	+
Coninckiidae	-	-	-	-	-	-	-	+	-	-
Cyatholaimidae	+	+	+	+	+	+	+	+	+	+
Desmodoridae	+	+	+	+	+	+	+	-	+	+
Desmoscolecidae	+	+	+	+	+	+	+	+	+	+
Diplopeltidae	-	+	+	+	-	-	-	-	+	+
Draconematidae	-	-	-	-	-	-	-	-	-	-
Enchelidiidae	+	+	+	+	-	+	-	+	+	+
Epsilonematidae	-	-	-	-	-	-	-	-	+	-
Ironidae	+	+	+	+	+	+	+	-	+	+
Lauratonematidae	-	+	-	-	-	-	-	-	-	-
Leptolaimidae	-	+	-	+	-	+	-	-	+	-
Leptosomatidae	+	-	-	-	-	-	-	-	+	+
Linhomoeidae	-	-	-	+	+	-	+	-	+	-
Meyliidae	+	+	+	-	+	+	-	+	+	+
Microlaimidae	-	+	+	+	+	+	-	-	+	+
Monhysteridae	+	+	+	+	+	+	+	+	+	+
Oncholaimidae	-	+	-	+	+	+	-	+	+	+
Oxystominidae	+	+	+	+	+	+	+	+	+	+
Pandolaimidae	-	-	-	-	-	-	-	-	+	+
Paramicrolaimidae	-	-	-	-	-	-	-	-	-	-
Phanodermatidae	+	+	+	-	+	+	-	-	-	+
Rhabdodemaniidae	-	-	-	-	-	-	-	-	-	-
Selachinematidae	+	+	+	+	+	+	+	+	+	+
Siphonolaimidae	-	-	-	-	-	-	-	-	-	-
Sphaerolaimidae	+	+	+	+	+	+	+	+	+	+
Symplocostomatidae	-	-	-	-	-	+	-	-	-	-
Thoracostomopsidae	+	+	+	-	+	+	-	-	-	-
Tripyloididae	-	-	+	+	-	-	-	-	+	+
Xyalidae	+	+	+	+	+	+	-	+	+	+

Table S7. Relative abundances >1% of nematode genera (% on total abundance) in the investigated morphologies, structures and in the up-/down-slope flanks of the mud waves and in the sediment inside and outside the trough and the scar.

Morphologies											
**Furrow**		**Sediment Waves**		**Trough**		**Scar**		**Other Depos. Struct.**		**Draped Sed.**	
**Genus**	**%**	**Genus**	**%**	**Genus**	**%**	**Genus**	**%**	**Genus**	**%**	**Genus**	**%**
Sabatieria	19	*Sabatieria*	26	*Sabatieria*	26	*Sabatieria*	28	*Sabatieria*	17	*Desmodora*	14
Desmoscolex	8	*Halalaimus*	8	*Desmodora*	8	*Acantholaimus*	12	*Halalaimus*	7	*Halomonhystera*	12
Sphaerolaimus	7	*Sphaerolaimus*	8	*Halalaimus*	5	*Sphaerolaimus*	8	*Desmodora*	7	*Sabatieria*	11
Amphimonhystrella	6	*Acantholaimus*	6	*Desmoscolex*	4	*Halalaimus*	7	*Desmoscolex*	6	*Halalaimus*	9
Desmodora	6	*Desmoscolex*	5	*Molgolaimus*	4	*Amphimonhystrella*	6	*Sphaerolaimus*	5	*Desmolorenzenia*	5
Halalaimus	6	*Syringolaimus*	4	*Amphimonhystrella*	3	*Linhystera*	5	*Acantholaimus*	4	*Karkinochromadora*	5
Acantholaimus	5	*Chromadorella*	4	*Desmolorenzenia*	3	*Desmolorenzenia*	4	*Amphimonhystrella*	4	*Oxystomina*	4
Chromadorella	5	*Amphimonhystrella*	3	*Linhystera*	3	*Richtersia*	4	*Richtersia*	4	*Sphaerolaimus*	4
Halichoanolaimus	3	*Desmodora*	3	*Sphaerolaimus*	3	*Desmoscolex*	3	*Tricoma*	3	*Endeolophos*	4
Spilophorella	3	*Halomonhystera*	2	*Richtersia*	3	*Halichoanolaimus*	3	*Chromadorella*	2	*Microlaimus*	3
Cobbia	3	*Pselionema*	2	*Syringolaimus*	3	*Oxystomina*	3	*Desmolorenzenia*	2	*Marylynnia*	3
Litinium	3	*Daptonema*	2	*Acantholaimus*	2	*Pselionema*	3	*Oxystomina*	2	*Syringolaimus*	3
Quadricoma	3	*Linhystera*	2	*Setosabatieria*	2	*Bathyeurystomina*	2	*Karkinochromadora*	2	*Richtersia*	2
Bathyeurystomina	2	*Tricoma*	2	*Halichoanolaimus*	1	*Karkinochromadora*	2	*Microlaimus*	2	*Linhystera*	2
Diplopeltoides	2	*Oxystomina*	2	*Oxystomina*	1	*Daptonema*	1	*Pselionema*	2	*Micoletzkyia*	2
Linhystera	2	*Halichoanolaimus*	2	*Quadricoma*	1	*Tricoma*	1	*Bathyeurystomina*	2	*Tricoma*	2
Bodonema	1	*Richtersia*	1	*Bathyeurystomina*	1			*Syringolaimus*	2	*Amphimonhystrella*	1
Molgolaimus	1	*Endeolophos*	1	*Pierrickia*	1			*Aponema*	2	*Daptonema*	1
Oxystomina	1	*Cobbia*	1	*Platycoma*	1			*Linhystera*	2	*Pselionema*	1
Pareudesmoscolex	1	*Karkinochromadora*	1	*Tricoma*	1			*Halichoanolaimus*	2	*Acantholaimus*	1
Richtersia	1	*Marylynnia*	1	*Actinonema*	1			*Marylynnia*	2	*Bathyeurystomina*	1
Syringolaimus	1			*Cobbia*	1			*Cobbia*	1	*Halichoanolaimus*	1
Tricoma	1			*Desmogerlachia*	1			*Metacyatholaimus*	1		
				Pselionema	1			*Stygodesmodora*	1		
				Spilophorella	1			*Endeolophos*	1		
				Viscosia	1			*Molgolaimus*	1		
								Phanoderma	1		

Table S7 (continue)

Structures									
**Sand Wave 1**		**Sand Wave 2**		**Mud Wave 1**		**Mud Wave 2**		**Mud Wave 3**	
**Genus**	**%**	**Genus**	**%**	**Genus**	**%**	**Genus**	**%**	**Genus**	**%**
Sabatieria	22	*Sabatieria*	28	*Sabatieria*	23	*Sabatieria*	29	*Sabatieria*	30
Halalaimus	9	*Syringolaimus*	10	*Desmoscolex*	7	*Sphaerolaimus*	16	*Halalaimus*	11
Chromadorella	6	*Halalaimus*	8	*Halalaimus*	7	*Halalaimus*	8	*Desmoscolex*	8
Cervonema	5	*Karkinochromadora*	4	*Sphaerolaimus*	6	*Acantholaimus*	6	*Acantholaimus*	8
Syringolaimus	5	*Desmodora*	4	*Acantholaimus*	6	*Syringolaimus*	4	*Sphaerolaimus*	7
Acantholaimus	5	*Linhystera*	4	*Halomonhystera*	5	*Amphimonhystrella*	4	*Chromadorella*	5
Desmodora	4	*Sphaerolaimus*	4	*Pselionema*	4	*Chromadorella*	3	*Amphimonhystrella*	3
Amphimonhystrella	3	*Halichoanolaimus*	3	*Tricoma*	3	*Southerniella*	3	*Endeolophos*	3
Daptonema	3	*Chromadorella*	2	*Oxystomina*	3	*Desmoscolex*	2	*Richtersia*	2
Karkinochromadora	3	*Daptonema*	2	*Syringolaimus*	3	*Halomonhystera*	2	*Syringolaimus*	2
Linhystera	3	*Desmolorenzenia*	2	*Desmodora*	3	*Halichoanolaimus*	2	*Aponema*	2
Cobbia	2	*Acantholaimus*	2	*Daptonema*	3	*Desmodora*	2	*Desmodora*	2
Desmoscolex	2	*Amphimonhystrella*	2	*Chromadorella*	2	*Endeolophos*	2	*Linhystera*	2
Sphaerolaimus	2	*Marylynnia*	2	*Amphimonhystrella*	2	*Richtersia*	2	*Tricoma*	2
Disconema	2	*Viscosia*	2	*Halichoanolaimus*	2	*Cobbia*	1	*Desmogerlachia*	1
Marylynnia	2	*Wieseria*	2	*Innocuonema*	2	*Oxystomina*	1	*Pselionema*	1
Oxystomina	2	*Aegialoalaimus*	1	*Linhystera*	2	*Pselionema*	1	*Daptonema*	1
Desmolorenzenia	2	*Desmoscolex*	1	*Bathyeurystomina*	2			*Halichoanolaimus*	1
Doliolaimus	2	*Elzalia*	1	*Marylynnia*	1				
Microlaimus	2	*Endeolophos*	1	*Cobbia*	1				
Euchromadora	1	*Halomonhystera*	1	*Richtersia*	1				
Micoletzkyia	1	*Metacyatholaimus*	1	*Quadricoma*	1				
Spirinia	1	*Metasphaerolaimus*	1						
		Micoletzkyia	1						
		Microlaimus	1						
		Molgolaimus	1						
		Oxystomina	1						
		Tricoma	1						

Table S7 (continue)

Structures					
**Dep. Structure 1**		**Dep. Structure 2**		**Dep. Structure 3**	
**Genus**	**%**	**Genus**	**%**	**Genus**	**%**
Sabatieria	20	*Sabatieria*	16	*Sabatieria*	15
Desmodora	16	*Sphaerolaimus*	7	*Desmoscolex*	10
Halalaimus	8	*Acantholaimus*	6	*Halalaimus*	9
Sphaerolaimus	7	*Desmoscolex*	5	*Desmodora*	6
Chromadorella	6	*Amphimonhystrella*	4	*Amphimonhystrella*	5
Stygodesmodora	5	*Richtersia*	4	*Richtersia*	4
Acantholaimus	2	*Tricoma*	4	*Acantholaimus*	4
Halichoanolaimus	2	*Halalaimus*	4	*Desmolorenzenia*	4
Karkinochromadora	2	*Aponema*	3	*Marylynnia*	4
Linhystera	2	*Metacyatholaimus*	3	*Tricoma*	4
Quadricoma	2	*Oxystomina*	3	*Bathyeurystomina*	3
Spilophorella	2	*Chromadorella*	3	*Microlaimus*	3
Aponema	2	*Cobbia*	3	*Endeolophos*	3
Desmolorenzenia	2	*Desmodora*	3	*Pselionema*	3
Desmoscolex	2	*Karkinochromadora*	3	*Molgolaimus*	2
Halomonhystera	2	*Syringolaimus*	3	*Karkinochromadora*	2
Microlaimus	2	*Pselionema*	2	*Linhystera*	2
Oxystomina	2	*Bathyeurystomina*	2	*Oxystomina*	2
Richtersia	2	*Desmolorenzenia*	2	*Phanoderma*	2
Syringolaimus	2	*Elzalia*	2	*Halichoanolaimus*	1
		Microlaimus	2	*Syringolaimus*	1
		Halichoanolaimus	1		
		Linhystera	1		
		Theristus	1		

Table S7 (continue)

Inside structures											
**Mud Wave 1**				**Mud Wave 2**				**Mud Wave 3**			
**Down**		**Up**		**Down**		**Up**		**Down**		**Up**	
**Genus**	**%**	**Genus**	**%**	**Genus**	**%**	**Genus**	**%**	**Genus**	**%**	**Genus**	**%**
Sabatieria	16	*Sabatieria*	29	*Sphaerolaimus*	27	*Sabatieria*	33	*Sabatieria*	34	*Sabatieria*	26
Desmoscolex	13	*Halalaimus*	9	*Sabatieria*	23	*Halalaimus*	10	*Halalaimus*	11	*Halalaimus*	10
Halomonhystera	9	*Acantholaimus*	7	*Chromadorella*	6	*Sphaerolaimus*	9	*Sphaerolaimus*	9	*Desmoscolex*	8
Sphaerolaimus	7	*Oxystomina*	6	*Halomonhystera*	6	*Acantholaimus*	8	*Acantholaimus*	8	*Acantholaimus*	7
Tricoma	6	*Sphaerolaimus*	5	*Southerniella*	6	*Amphimonhystrella*	6	*Desmoscolex*	8	*Chromadorella*	6
Pselionema	5	*Syringolaimus*	5	*Halalaimus*	5	*Syringolaimus*	6	*Chromadorella*	3	*Sphaerolaimus*	5
Halalaimus	5	*Daptonema*	3	*Acantholaimus*	3	*Richtersia*	3	*Amphimonhystrella*	3	*Richtersia*	4
Acantholaimus	5	*Desmodora*	3	*Desmodora*	2	*Desmoscolex*	2	*Aponema*	1	*Endeolophos*	4
Innocuonema	4	*Cobbia*	3	*Desmoscolex*	2	*Halichoanolaimus*	2	*Daptonema*	1	*Amphimonhystrella*	3
Chromadorella	3	*Halichoanolaimus*	2	*Syringolaimus*	2	*Endeolophos*	2	*Desmodora*	1	*Desmogerlachia*	2
Desmodora	3	*Pselionema*	2	*Halichoanolaimus*	2	*Chromadorella*	2	*Endeolophos*	1	*Linhystera*	2
Daptonema	2	*Amphimonhystrella*	2	*Pselionema*	2	*Cobbia*	2	*Halichoanolaimus*	1	*Syringolaimus*	2
Quadricoma	2	*Bathyeurystomina*	2	*Amphimonhystrella*	1	*Desmodora*	2	*Syringolaimus*	1	*Aponema*	2
Amphimonhystrella	2	*Chromadorella*	2	*Cobbia*	1	*Bathyeurystomina*	1	*Elzalia*	1	*Desmodora*	2
Linhystera	2	*Desmoscolex*	2	*Endeolophos*	1	*Karkinochromadora*	1	*Karkinochromadora*	1	*Tricoma*	2
Marylynnia	2	*Linhystera*	2	*Oxystomina*	1	*Oxystomina*	1	*Marylynnia*	1	*Pselionema*	2
Paracanthonchus	2	*Richtersia*	2	*Tricoma*	1			*Pselionema*	1	*Leptolaimus*	1
Bathyeurystomina	1	*Vasostoma*	2					*Tricoma*	1	*Viscosia*	1
Halichoanolaimus	1	*Marylynnia*	1								
Camacolaimus	1	*Tricoma*	1								
Molgolaimus	1										
Richtersia	1										

Table S7 (continue)

Inside structures

Trough				Scar			
**Inside**		**Outside**		**Inside**		**Outside**	
**Genus**	**%**	**Genus**	**%**	**Genus**	**%**	**Genus**	**%**
Sabatieria	23	*Sabatieria*	30	*Sabatieria*	36	*Sabatieria*	25
Molgolaimus	7	*Desmodora*	10	*Sphaerolaimus*	14	*Acantholaimus*	14
Desmodora	6	*Desmoscolex*	6	*Desmoscolex*	9	*Linhystera*	7
Desmolorenzenia	6	*Halalaimus*	5	*Halalaimus*	9	*Desmolorenzenia*	6
Halalaimus	5	*Amphimonhystrella*	4	*Acantholaimus*	6	*Halalaimus*	6
Linhystera	4	*Richtersia*	3	*Amphimonhystrella*	6	*Amphimonhystrella*	5
Sphaerolaimus	4	*Syringolaimus*	3	*Halichoanolaimus*	4	*Richtersia*	5
Amphimonhystrella	3	*Acantholaimus*	3	*Pselionema*	4	*Sphaerolaimus*	5
Desmoscolex	3	*Linhystera*	3	*Cervonema*	3	*Oxystomina*	4
Oxystomina	3	*Setosabatieria*	3	*Desmodora*	3	*Bathyeurystomina*	2
Richtersia	3	*Sphaerolaimus*	3	*Disconema*	1	*Halichoanolaimus*	2
Syringolaimus	3	*Actinonema*	2	*Marylynnia*	1	*Karkinochromadora*	2
Acantholaimus	2	*Halichoanolaimus*	2	*Richtersia*	1	*Daptonema*	2
Cobbia	2	*Platycoma*	2	*Setosabatieria*	1	*Pselionema*	2
Pierrickia	2	*Pselionema*	2	*Syringolaimus*	1	*Tricoma*	2
Amphimonhystera	1	*Spilophorella*	2			*Acatarjania*	1
Araeolaimus	1	*Endeolophos*	2			*Cobbia*	1
Bathyeurystomina	1	*Quadricoma*	2			*Desmoscolex*	1
Desmogerlachia	1	*Alaimella*	1			*Euchromadora*	1
Quadricoma	1	*Bathyeurystomina*	1			*Viscosia*	1
Setosabatieria	1	*Campylaimus*	1			*Aegialoalaimus*	1
Tricoma	1	*Desmolorenzenia*	1			*Anoplostoma*	1
		Filoncholaimus	1			*Coninckia*	1
		Karkinochromadora	1			*Diplopeltoides*	1
		Pandolaimus	1			*Marylynnia*	1
		Pareurystomina	1				
		Phanodermopsis	1				
		Tricoma	1				
		Viscosia	1				

Figure S1. Vertical meiofaunal distribution in sediment layers of the different seafloor morphologies. Reported are nematodes, copepods, tardigrades and others (polychaetes, ostracods, gastrotrichs, amphipods, isopods, acarines, priapulids, decapods, gastropods and kinorhynchs).

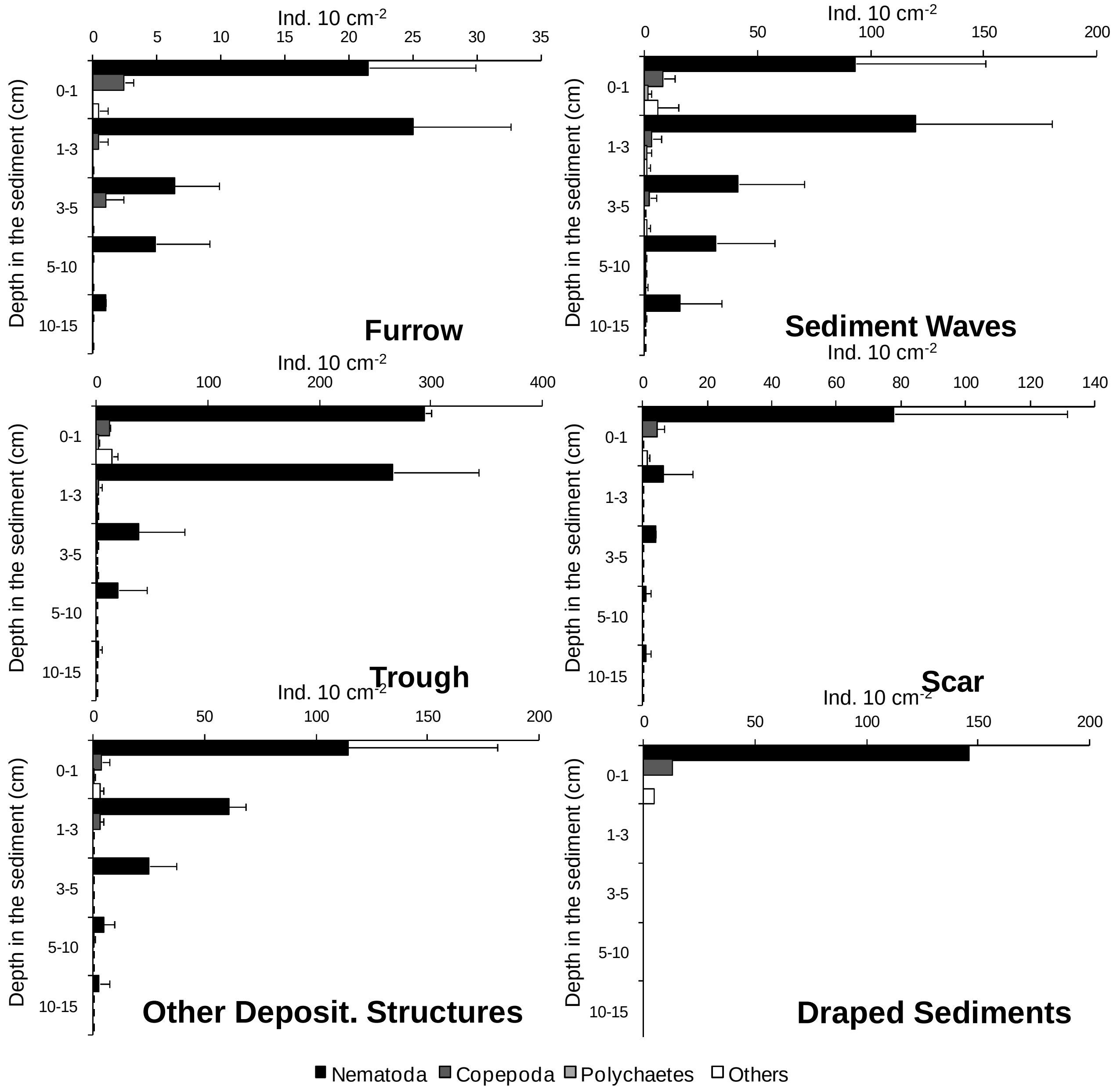

Supplement: Supplementary Information [file srep26352-s1.pdf]
